# Pseudorabies virus tegument protein UL13 recruits RNF5 to inhibit STING-mediated antiviral immunity

**DOI:** 10.1371/journal.ppat.1010544

**Published:** 2022-05-18

**Authors:** Zhengjie Kong, Hongyan Yin, Fan Wang, Zhen Liu, Xiaohan Luan, Lei Sun, Wenjun Liu, Yingli Shang

**Affiliations:** 1 Department of Preventive Veterinary Medicine, College of Veterinary Medicine, Shandong Agricultural University, Taian, Shandong, China; 2 Shandong Provincial Key Laboratory of Animal Biotechnology and Disease Control and Prevention, Shandong Agricultural University, Taian, Shandong, China; 3 CAS Key Laboratory of Pathogenic Microbiology and Immunology, Institute of Microbiology, Chinese Academy of Sciences, Beijing, China; 4 University of Chinese Academy of Sciences, Beijing, China; 5 Institute of Immunology, Shandong Agricultural University, Taian, Shandong, China; Oregon Health and Sciences University, UNITED STATES

## Abstract

Pseudorabies virus (PRV) has evolved various immune evasion mechanisms that target host antiviral immune responses. However, it is unclear whether and how PRV encoded proteins modulate the cGAS-STING axis for immune evasion. Here, we show that PRV tegument protein UL13 inhibits STING-mediated antiviral signaling via regulation of STING stability. Mechanistically, UL13 interacts with the CDN domain of STING and recruits the E3 ligase RING-finger protein 5 (RNF5) to promote K27-/K29-linked ubiquitination and degradation of STING. Consequently, deficiency of RNF5 enhances host antiviral immune responses triggered by PRV infection. In addition, mutant PRV lacking UL13 impaired in antagonism of STING-mediated production of type I IFNs and shows attenuated pathogenicity in mice. Our findings suggest that PRV UL13 functions as an antagonist of IFN signaling via a novel mechanism by targeting STING to persistently evade host antiviral responses.

## Author summary

Induction of type I interferons mediated by cGAS-STING axis is critical for host against DNA virus infection whereas herpesviruses employ multiple strategies to antagonize this signaling pathway for immune evasion. Herein, our findings provide strong evidence that PRV tegument protein UL13 functions as a suppressor of STING-mediated antiviral response via recruitment of E3 ligase RNF5 to induce K27-/K29-linked ubiquitination and degradation of STING. Therefore, our study reveals a novel evasion strategy of PRV against host defense and suggests UL13 could be a promising target for development of gene-deleted vaccine for pseudorabies.

## Introduction

The innate immune system serves as the first line of host defense against invading pathogens. Upon viral infection, host cells detect structurally conserved pathogen-associated molecular patterns and rapidly launch a series of signaling events that subsequently lead to production of type I interferons (IFNs) and other antiviral factors [1]. Among multiple DNA sensors, the cyclic GMP-AMP synthase (cGAS) has been demonstrated as a general sensor to recognize cytosolic DNA in response to DNA viral infection [2]. cGAS recognizes double-stranded DNA (dsDNA) and synthesizes the second messenger cyclic GMP-AMP (cGAMP), which binds and activates the endoplasmic reticulum (ER)-associated adaptor protein stimulator of interferon gene (STING) [3]. STING then dimerizes, oligomerizes, and recruits TANK-binding kinase (TBK1) [4], which results in TBK1 dimerization and phosphorylation. The cascade reactions then promote TBK kinase activity and phosphorylate the transcription factor interferon regulatory factor (IRF3), leading to IRF3 nuclear transduction and IFN production [5–7].

As cGAS-STING axis is crucial for host antiviral responses, viruses have evolved various strategies to antagonize this signaling pathway for immune evasion [8]. For example, it has been reported that multiple tegument proteins of DNA viruses play critical roles in regulation of innate immune responses via targeting cGAS-STING axis [9]. The herpes simplex virus 1 (HSV-1) protein ICP27 interacts with STING-TBK1 complex to inhibit IRF3 phosphorylation [10] and the tegument proteins UL41 and UL46 of HSV-1 directly degrade cGAS mRNA or inhibit TBK1 activation respectively [11,12]. Similarly, human cytomegalovirus (HCMV) tegument protein UL82 was reported to impair the trafficking of STING and the recruitment of TBK1 or IRF3 to STING [13]. Moreover, HCMV US9 was confirmed to disrupt STING-TBK1 association and block IRF3 nuclear translocation [14]. Despite these findings, the roles and mechanisms of herpesvirus-encoded tegument proteins targeting cGAS-STING pathways remain largely unclear.

Pseudorabies virus (PRV) belongs to the number of the alphaherpesvirus subfamily, which is also known as suid herpesvirus 1 or Aujeszky’s disease virus and infects a broad range of vertebrates including its natural host swine [15]. PRV infection in swine can result in devastating disease and economic losses worldwide [16]. Particularly, recent studies have shown that variant PRV can directly infect humans, which leads to severe damage in nervous and respiratory systems, raising the concern of PRV cross-species transmission [17,18]. PRV has a large linear double-stranded DNA genome that encodes more than 70 functional proteins [19]. Recently, multiple PRV-encoded proteins were reported to inhibit host innate antiviral response which facilitate replication and latent viral infection [20]. For instance, it has been shown that the viral glycoprotein gE/gI complex reduces the phosphorylation of ERK1/2 to suppress production of type I IFNs in plasmacytoid dendritic cells [21]. PRV UL50 suppresses type I IFN signaling by promoting lysosomal degradation of IFNAR1 [22]. In addition, PRV US3 inhibits IFN signaling by promoting degradation of the host protein Bclaf1 [23]. However, whether PRV proteins target the cGAS-STING axis for immune evasion is unknown.

In this study, we identified that PRV tegument protein UL13 as the suppressor of STING-mediated signaling to inhibit IFN production and antiviral response during RPV infection. UL13 interacts with STING in cytosol and impairs STING protein stability whereas knockdown or deficiency of UL13 enhances the protein stability of STING. Particularly, UL13 recruits the E3 ubiquitin ligase RING finger protein 5 (RNF5) to promote K27-/K29-linked ubiquitination and degradation of STING thereby impairing STING-mediated production of type I IFNs and expression of other downstream antiviral factors. Our findings thus identified a new strategy and mechanism for PRV to evade STING-mediated antiviral immune responses via recruitment of RNF5 to modulate STING stability.

## Results

### UL13 Inhibits STING-Mediated Signaling

**Fig 1:**
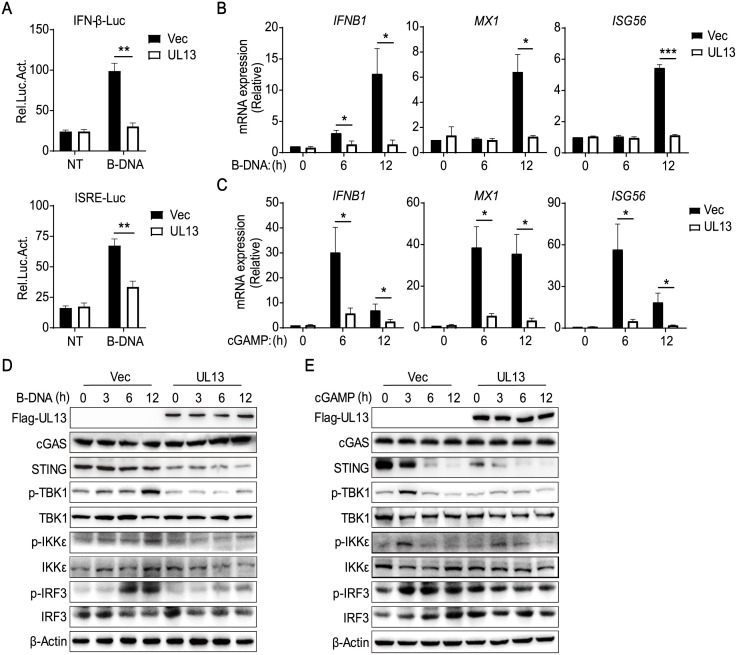
Identification of PRV UL13 as an inhibitor of cGAS-STING signaling. (A) Luciferase activities in UL13 PK-15 or control PK-15 cells cotransfected with IFN-β-luc reporter or ISRE-luc reporter. Twenty-four hours after transfection, cells were left untreated or stimulated with B-DNA (2 μg/ml) for 12 h, and cell lysates were analyzed for luciferase activity. (B and C) qPCR analysis of *IFNB1* and downstream ISGs (*MX1*, *ISG56*) mRNA expression in UL13-PK-15 cells and control cells stimulated with B-DNA (1 μg/ml) (B) or cGAMP (1 μg/ml) (C) for the indicated times. (D and E) Immunoblotting analysis of cGAS, STING, phosphorylated (Ser172)- and total TBK1 and IKKε, phosphorylated (Ser396)- and total IRF3 in whole-cell lysates of UL13-PK-15 cells and control cells stimulated for the various times (above lanes) with B-DNA (1 μg/ml) (D) or cGAMP (1 μg/ml) (E). Data are pooled from three independent experiments (A-C, mean ± SD) or representative of three independent experiments (D, E). *p < 0.05, **p < 0.01 and ***p < 0.001 (Student’s *t* test).

It has been demonstrated that the cGAS-STING axis plays a critical role in the induction of type I IFNs in response to herpesvirus infection [24]. To identify PRV tegument proteins that target STING-mediated signaling, we constructed 15 expression clones encoding key PRV tegument proteins and performed luciferase assays to screen for tegument proteins that could regulate STING-mediated activation of IFN-β promoter in PK-15 cells. Of note, we identified PRV UL13 and US3, two protein kinases of PRV, dramatically inhibited the activity of IFN-β promoter upon B-DNA Poly (dA:dT) stimulation (S1A Fig), indicating that UL13 and US3 function as suppressors of IFN expression. Next, we focused on UL13 because the suppression of IFN mediated by UL13 is the most striking and reliable. To further investigate the role of UL13 on IFN expression, we established stable PK-15 cells ectopically expressing UL13 (UL13-PK-15 cells) by lentiviral-mediated transduction [13,25]. The suppressive role of UL13 on DNA-induced activation of IFN-β promoter and the interferon-stimulated response element (ISRE) were verified by luciferase assays in UL13-PK-15 cells and empty vector-transduced control cells (Fig 1A). Also, upon B-DNA Poly (dA:dT) stimulation, UL13-PK-15 cells showed impaired gene expression of *IFNB1* and downstream ISGs, including *MX1*, *MX2*, *ISG15*, *ISG54*, *ISG56*, and *OSA1b*, compared with control cells (Fig 1B and S1B Fig). These data confirm that UL13 acts as an antagonist of type I IFNs.

The transfected cytosolic DNA is mainly recognized by cGAS and activated cGAS triggers the production of cGAMP, which binds and activates the adaptor protein STING to elicit innate immune responses [26]. To know whether UL13 targets cGAS-STING signaling pathway, we examined the effect of UL13 on cGAMP-mediated innate immune responses. Similarly, UL13 suppressed cGAMP-induced transcription of *IFNB1* and ISGs in PK-15 cells (Fig 1C and S1C Fig), indicating that UL13 likely targets the downstream of cGAS to suppress DNA-mediated immune responses. Consequently, UL13 inhibited the phosphorylation of TBK1, IKKε, and IRF3 induced by transfected B-DNA (Fig 1D and S1D Fig), or cGAMP particularly at the early time point (Fig 1E and S1E Fig), suggesting that UL13 negatively regulates activation of cGAS-STING-mediated signaling. Notably, UL13 expression markedly decreased the protein level of STING without affecting protein expression of cGAS (Fig 1D and 1E), indicating that UL13 possibly represses STING-mediated innate immune responses via modulating STING expression. Notably, while UL13 expression inhibited phosphorylation of TBK1, IKKε, and IRF3 induced by B-DNA or cGAMP stimulation, UL13 did not alter total protein levels of TBK1, total IKKε and IRF3 (Fig 1D and 1E). Taken together, these findings demonstrate that PRV tegument protein UL13 negatively regulates STING-mediated antiviral immune responses.

### UL13-deficiency heightens innate antiviral response to PRV

**Fig 2:**
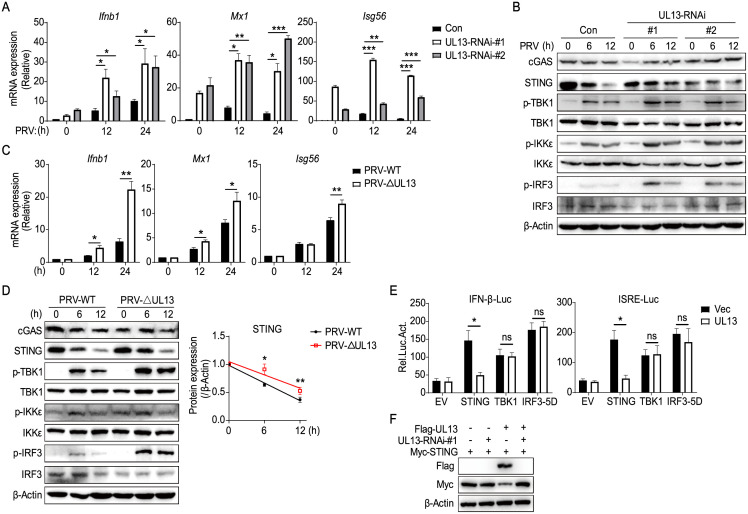
Knockdown of UL13 increases PRV-triggered antiviral responses. (A) qPCR analysis of PRV-induced transcription of *Ifnb1* and downstream genes (*Mx1*, *Isg56*) in UL13-RNAi stable mouse embryonic fibroblasts (MEFs) and control cells infected with PRV (MOI = 1) infection for the indicated periods. (B) Immunoblotting analysis of cGAS, STING, and phosphorylation of downstream components in UL13-RNAi MEFs and control cells infected with PRV (MOI = 1) for the indicated times. (C and D) qPCR analysis of transcription of *Ifnb1* and downstream genes (*Mx1*, *Isg56*) (C) or immunoblotting analysis of cGAS, STING, and phosphorylation of downstream components (D) in MEFs infected with wild type PRV (strain Bartha-K61, PRV-WT, MOI = 1) or UL13-deficient PRV (PRV-ΔUL13, MOI = 1) for the indicated times. Cumulative results for densitometric quantitation of STING were normalized relative to the levels of 0 min conditions (D, right). (E) Luciferase activities in UL13-PK-15 cells and control PK-15 cells cotransfected with IFN-β-luc reporter or ISRE-luc reporter and plasmids encoding STING, TBK1, or IRF3-5D or empty vector as indicated. Twenty-four hours after transfection, cell lysates were analyzed for luciferase activity. (F) Immunoblotting analysis of Flag-UL13 and Myc-STING expression in HEK293T cells cotransfected with Myc-STING, Flag-UL13 or UL13 shRNA plasmids as indicated for 24 h. Data are pooled from three independent experiments (A, C, D, right and E, mean ± SD) or representative of three (B, D, left) or two (F) independent experiments. *p < 0.05, **p < 0.01 and ***p < 0.001, ns, not significant (Student’s *t* test).

Having known that UL13 expression inhibits DNA-triggered induction of IFN and downstream antiviral genes, we next investigated the effect of UL13 knockdown on host antiviral immune responses. To do this, we firstly constructed two UL13-short hairpin RNAs (shRNAs) plasmids and confirmed that both UL13 shRNAs can specifically suppress protein expression of Flag-tagged UL13 or gene expression of PRV-expressed UL13 in HEK293T cells (S2A and S2B Fig) [27,28]. Considering that HEK293T cells express very low levels of STING we then generated MEFs stably expressing UL13-shRNAs or a control shRNA respectively [29]. qPCR analysis showed that MEFs stably expressing UL13-shRNAs strikingly reduced mRNA level of PRV-expressed UL13 (S2C Fig). Compared with the cells transduced with control shRNA, PRV-induced transcription of *Ifnb1*, *Mx1*, and *Isg56* was increased in UL13-knockdown cells in different periods post-infection (Fig 2A), suggesting that knockdown of UL13 enhances PRV-triggered antiviral responses. Moreover, knockdown of UL13 in MEFs also enhanced phosphorylation of TBK1, IKKε, and IRF3, the downstream components of STING, and stabilized the protein levels of STING at 6 and 12 h post-infection of PRV (Fig 2B and S2D Fig). In contrast, knockdown of UL13 did not alter the proteins levels of cGAS and total TBK1, IKKε, and IRF3 in MEFs (Fig 2B). These results suggest that knockdown of UL13 increases PRV-triggered antiviral immune responses via modulation of STING protein levels. To further confirm the regulatory functions of UL13, we generated UL13-deficient PRV by CRISPR/Cas9 technology. The deletion mutation of UL13 in mutant PRV genome was verified by PCR assay with specific primers following DNA sequencing (S2E Fig). We first determined the effects of deficiency of UL13 on viral replication and observed similar propagation between wild-type or UL13-deficient PRV (PRV-ΔUL13) in MEFs (S2F Fig), indicating that UL13 is nonessential for PRV replication. Next, we investigated the expression of downstream antiviral genes in MEFs induced by wild-type or UL13-deficient PRV. The results showed that induction of *Ifnb1* and downstream ISGs, including *Mx1*, *Mx2*, *Isg15*, *Isg56*, and *Oas1b*, mediated by PRV-ΔUL13 was significantly higher than those induced by wild-type PRV in MEFs (Fig 2C and S2G Fig). In line with it, phosphorylation of TBK1, IKKε, and IRF3 induced by PRV-ΔUL13 was markedly increased in comparison to that induced by wild-type PRV (Fig 2D, left). Meanwhile, the protein levels of STING decreased slower following infection with PRV-ΔUL13 compared to wild-type PRV (Fig 2D, right; S2H Fig). To know whether UL13 acts at the level of STING or its downstream components, luciferase assays were then performed by overexpression of STING, TBK1, IRF3-5D with IFN-β or ISRE luciferase in UL13-PK-15 and control cells. The results showed that UL13 expression significantly reduced STING-mediated activation of IFN-β and ISRE promoters but did not affect TBK1- or IRF3-5D-mediated activation of IFN-β and ISRE promoters (Fig 2E), suggesting that UL13 indeed acts at the level of STING. Additionally, UL13-mediated decrease of STING protein level was restored in presence of UL13 shRNA in HEK293T cell co-transfection system (Fig 2F), indicating that knockdown of UL13 enhances protein expression of STING. Collectively, these results suggest that UL13 plays a direct role in PRV evasion of innate antiviral response via targeting STING.

### UL13 interacts with STING and impairs its protein expression

**Fig 3:**
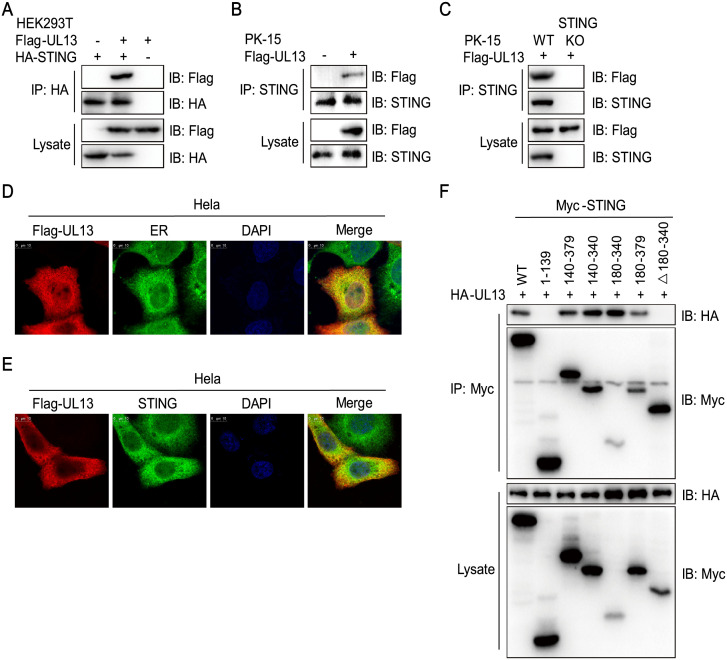
UL13 associates with STING. (A-C) Immunoblotting (IB) analysis of indicated proteins in immunoprecipitated (IP) samples and whole-cell lysates of HEK293T cells transfected with Flag-UL13 and HA-STING (A), whole-cell lysates of PK-15 cells transfected with Flag-UL13 (B), or STING-deficient PK-15 cells transfected with Flag-UL13 (C). Anti-HA (A) or anti-STING (B, C) immunoprecipitates were analyzed by immunoblotting with anti-Flag antibodies. Levels of the transfected proteins were analyzed by immunoblotting with anti-HA (A), anti-STING (B, C), or anti-Flag (A-C) antibodies. (D) Colocalization of UL13 and STING. Hela cells were transfected with Flag-UL13 as indicated for 24 h before confocal microscopy. Endogenous STING was labeled by anti-STING antibody. (E) Colocalization of UL13 and endoplasmic reticulum (ER). Hela cells were transfected with Flag-UL13 for 24 h before confocal microscopy. ER was labeled with anti-Calreticulin antibody. (F) Immunoblotting analysis of UL13 and STING in immunoprecipitated samples or whole-cell lysates of HEK293T cells transfected with HA-UL13 and full length or truncated Myc-STING. Anti-Myc immunoprecipitates were analyzed by immunoblotting with an anti-HA or anti-Myc antibody. Levels of the transfected proteins were analyzed by immunoblotting with an anti-HA or anti-Myc antibody. Scale bars, 10 μm. Data are representative of two independent experiments (A-F).

Next, we investigated the molecular mechanisms of UL13 on negative regulation of STING-mediated innate antiviral response. Compared with control PK-15 cells, UL13 expression did not affect mRNA expression of STING in UL13-PK-15 cells (S3A Fig). qPCR analysis also showed that UL13 did not affect mRNA expression of STING in MEFs infected by PRV (S3B Fig). According to these data, we next determine whether UL13 impairs the protein expression of STING at post-transcriptional level. Indeed, transient transfection and coimmunoprecipitation experiments indicated that UL13 was associated with STING in HEK293T cells (Fig 3A and S4A Fig). Confocal microscopy analysis also showed that UL13 co-localized with STING in Hela cells (S4B Fig), which strongly indicated that UL13 could associate with STING. Moreover, coimmunoprecipitation experiments also indicated that UL13 could bind to endogenous STING in PK-15 cells (Fig 3B and 3C). In addition, UL13 localized at endoplasmic reticulum (ER) and co-localized with endogenous STING (Fig 3D and 3E), confirming that UL13 interacts with STING. Domain-mapping experiments showed that the cyclic dinucleotides (CDN)-binding region of STING (aa 180–340) was sufficient for their interaction (Fig 3F) [30], indicating that the CDN domain of STING is critical for their interaction. Together, these results suggest that UL13 and STING interaction may be responsible for STING protein regulation.

### UL13 enhances K27-/K29-linked ubiquitination of STING

**Fig 4:**
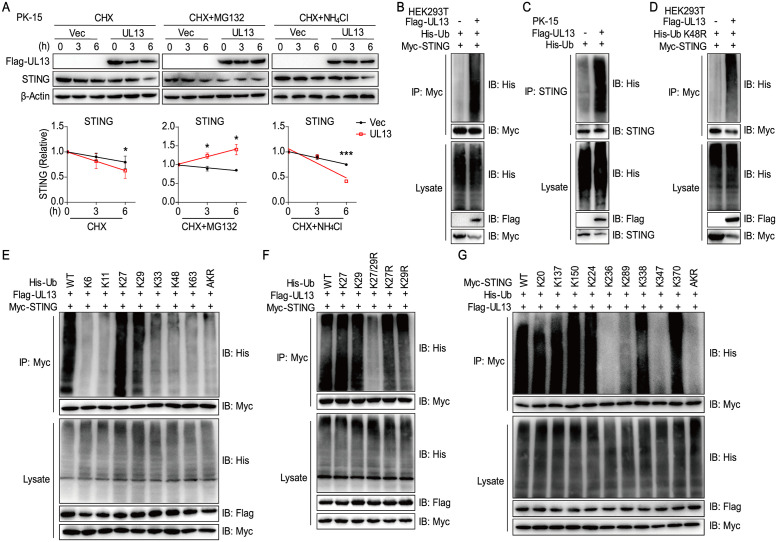
UL13 targets STING for K27-/K29-linked ubiquitination and degradation. (A) Immunoblotting analysis of STING protein expression in whole-cell lysates of PK-15 cells transfected with UL13 expression plasmid or empty vector following treatment with cycloheximide CHX (left), MG-132 (middle), or NH_4_Cl (right) (10 μM) for the indicated times, respectively. Densitometric quantitation of STING was normalized relative to the levels of 0 h conditions (A, lower). (B-D) Immunoprecipitation analysis of HEK293T cells expressing His-Ubiquitin (Ub) and Myc-STING together with or without Flag-UL13 (B), PK-15 cells expressing His-Ub with or without Flag-UL13 (C), or HEK293T cells expressing His-Ub K48R mutant (Lys-to-Arg mutant at position 48 only) and Myc-STING together with or without Flag-UL13 (D). (E) Immunoprecipitation analysis of HEK293T cells expressing Flag-UL13 and Myc-STING together with wild type (WT) His-Ub or His-Ub mutants at K6, K11, K27, K29, K33, K48, and K63 only; or Lys-to-Arg mutants of all Ub lysines, AKR) as indicated. (F) Immunoprecipitation analysis of HEK293T cells expressing Flag-UL13 and Myc-STING together with WT His-Ub, His-Ub at K27 or K29 only or His-K27R, K29R, or K27/29R only mutants as indicated. (G) Immunoprecipitation analysis of HEK293T cells expressing Flag-UL13 and His-Ub together with wild type (WT) Myc-STING or Myc-STING mutants (K20, K137, K150, K224, K236, K289, K338, K347, and K370 only; or Lys-to-Arg mutants of all STING lysines, AKR) as indicated. Anti-Myc (B, D-G) or anti-STING (C) immunoprecipitates were analyzed by immunoblotting with an anti-His, anti-Myc or anti-STING antibody. Levels of the transfected proteins were analyzed by immunoblotting with anti-Flag, anti-His, anti-Myc or anti-STING antibody as indicated. Data are representative of three (A, upper) or two (B-G) independent experiments, or pooled from three independent experiments (A, lower). *p < 0.05, ***p < 0.001 (Student’s *t* test).

Having known that UL13 interacts with STING and modulates the protein expression of STING, we next determine how UL13 affects the protein levels of STING. First, PK-15 cells transfected with UL13 showed a significant reduction of STING protein levels compared with empty vector-transduced PK-15 cells when cells were treated with cycloheximide (CHX), a protein synthesis inhibitor, indicating that UL13 controls the stability of STING (Fig 4A, left). To further investigate the mechanisms of UL13-induced STING protein degradation, we treated PK-15 cells with inhibitors for proteasome or lysosomal degradation pathways [31]. The proteasome inhibitor MG-132 blocked STING degradation after termination of protein synthesis by CHX in UL13-PK-15 cells compared to that of control cells (Fig 4A, middle). By contrast, the lysosomal inhibitor ammonium chloride (NH_4_Cl) did not retard STING degradation in UL13-PK-15 cells after CHX treatment (Fig 4A, right). These results indicate that UL13 promotes the degradation of STING protein via proteasome pathway. Next, we co-expressed Flag-UL13, His-ubiquitin (His-Ub), and Myc-STING in HEK293T cells to determine whether UL13 affects the ubiquitination of STING. As expected, expression of UL13 substantially increased STING ubiquitination (Fig 4B). Moreover, UL13 expression also enhanced the ubiquitination of endogenous STING in PK-15 cells (Fig 4C). These data demonstrate that UL13 promotes STING ubiquitination for degradation.

The K48-linked ubiquitin chain is thought to be the main signal for proteasome-mediated degradation [32] and multiple E3 ligases were reported to induce K48-linked ubiquitination and degradation of STING [33–35]. Hence, we sought to know whether UL13 promotes K48-linked ubiquitination of STING. Surprisingly, UL13 still promoted the ubiquitination of STING in HEK293T cells when a ubiquitin mutant at position 48 (a lysine residue at position 48 was substituted for arginine, K48R) was expressed (Fig 4D), which excluded To characterize the linkage of ubiquitin chain on STING modulated by UL13, we generated a series of ubiquitin mutants containing a lysine residue only at position 6, 11, 27, 29, 33, 48, or 63 alone, or all lysine residues to arginine mutant (AKR), in which all lysine residues were substituted for arginine. Immunoprecipitation assays showed that expression of UL13 predominantly induced the ubiquitination of STING in the presence of ubiquitin mutants containing a lysine residue at position 27 or 29 (Fig 4E), indicating that UL13 particularly facilitates K27-/K29-linked polyubiquitination of STING. Furthermore, UL13 failed to enhance STING polyubiquitination when all the lysines of ubiquitin were substituted with arginine (AKR) or in K27/K29R mutants, in which lysines at positions 27 and 29 were mutated to arginines (Fig 4F), confirming that UL13 promotes K27-/K29-linked of STING polyubiquitination. To map the ubiquitination sites on STING, we further generated a series of STING mutants containing one lysine residue only at position 20, 137, 150, 224, 236, 289, 338, 347, or 370, or all lysine residues to arginine mutant (STING-AKR). Immunoprecipitation assays showed expression of UL13 predominantly induced the ubiquitination of STING at position 20, 137, 150, 224, 338, or 370 (Fig 4G), indicating that these six lysines (including K20, 137, 150, 224, 338, and 370) of STING are responsible for UL13-mediated polyubiquitination of STING. Taken together, these data demonstrated that UL13 induced K27-/K29-linked polyubiquitination of STING at multiple lysines for degradation.

### UL13 Recruits RNF5 to initiate the K27-/K29-linked ubiquitination of STING

**Fig 5:**
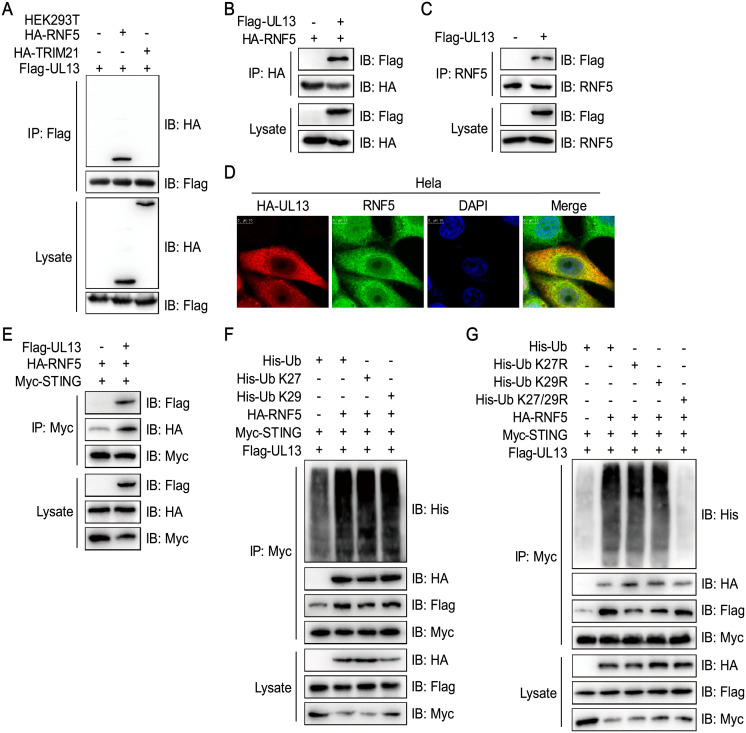
UL13 recruits E3 ligase RNF5 to induce STING ubiquitination degradation. (A to C) Immunoprecipitation analysis of HEK293T cells expressing Flag-UL13 together with HA-TRIM21 and HA-RNF5 (A) or HA-RNF5 (B) or Flag-UL13 alone (C). Anti-Flag (A), anti-HA (B), or anti-RNF5 (C) immunoprecipitates were analyzed by immunoblotting with anti-HA (A) and anti-Flag (B, C) antibodies. Levels of the transfected proteins were analyzed by immunoblotting with anti-Flag and anti-HA antibodies (A, B) or anti-RNF5 antibodies. (D) Colocalization of UL13 and RNF5 in Hela cells. Cells were transfected with HA-UL13 for 24 h before confocal microscopy. Endogenous RNF5 was labeled by anti-RNF5 antibody. (E) Immunoprecipitation analysis of HEK293T cells expressing Myc-STING and HA-RNF5 with or without Flag-UL13. Anti-Myc immunoprecipitates were analyzed by immunoblotting with anti-HA and anti-Flag antibodies. The levels of the transfected proteins were analyzed by immunoblotting with anti-Flag, anti-HA, or anti-Myc antibodies. (F, G) Immunoprecipitation analysis of HEK293T cells expressing Flag-UL13, Myc-STING, and His-Ub (WT, K27, or K29 only) with or without HA-RNF5 (F) or HEK293T cells expressing Flag-UL13 and Myc-STING together with His-Ub (WT, K27R, K29R, or K27/29R only mutants) as indicated with or without HA-RNF5 (G). Anti-Myc immunoprecipitates were analyzed by immunoblotting with anti-His, anti-Flag, and anti-HA antibodies (F, G). The levels of the transfected proteins were analyzed by immunoblotting with anti-Flag, anti-HA, or anti-Myc antibodies. Scale bars, 10 μm (D). Data are representative of two independent experiments (A-G).

Previous studies suggested that the E3 ubiquitin ligases RNF5, TRIM29, and TRIM30α are responsible for ubiquitination and degradation of STING [33–35]. Since UL13 induced K27-/K29-linked ubiquitination of STING, we next wanted to identify the possible ubiquitin enzymes that are involved in this process. Therefore, we performed immunoprecipitation by transfecting with HA-STING and Flag-UL13 in HEK293T cells. Mass spectrometry analysis showed that tripartite motif-containing protein 21 (TRIM21), HECT, UBA and WWE domain-containing protein 1 (HUWE1), and RNF5, three known E3 ligases, are likely associated with STING (S5A Fig). It has been shown that TRIM21 and RNF5 are related to regulation of cytoplasmic DNA recognition signaling pathway [33,36]. Therefore, we examined the binding of TRIM21 and RNF5 to UL13. Immunoprecipitation assays showed that UL13 indeed interacted with HA-tagged RNF5 but not TRIM21 in HEK293T cells, indicating that UL13 possibly facilitates ubiquitination of STING through interaction with the E3 ligase RNF5 (Fig 5A and 5B). The interaction and colocalization of UL13 with endogenous RNF5 were reproducible in HEK 293T and Hela cells (Fig 5C and 5D), supporting that UL13 interacts with RNF5 endogenously. Accordingly, expression of UL13 promoted the binding of RNF5 to STING (Fig 5E), implying that UL13 functionally conduces to the interaction between E3 ligase RNF5 and STING. Indeed, expression of RNF5 remarkably increased UL13-related K27-/K29-linked ubiquitination and degradation of STING and enhanced the interaction between UL13 and STING (Fig 5F). Conversely, RNF5 expression failed to elevate UL13-mediated STING polyubiquitination when a ubiquitin K27/K29R mutant was introduced (Fig 5G). Together, these results suggest that UL13 recruits the E3 ligase RNF5 to induce STING K27-/K29-linked ubiquitination and degradation.

### RNF5 deficiency retards STING ubiquitination and potentiates PRV-triggered antiviral response

**Fig 6:**
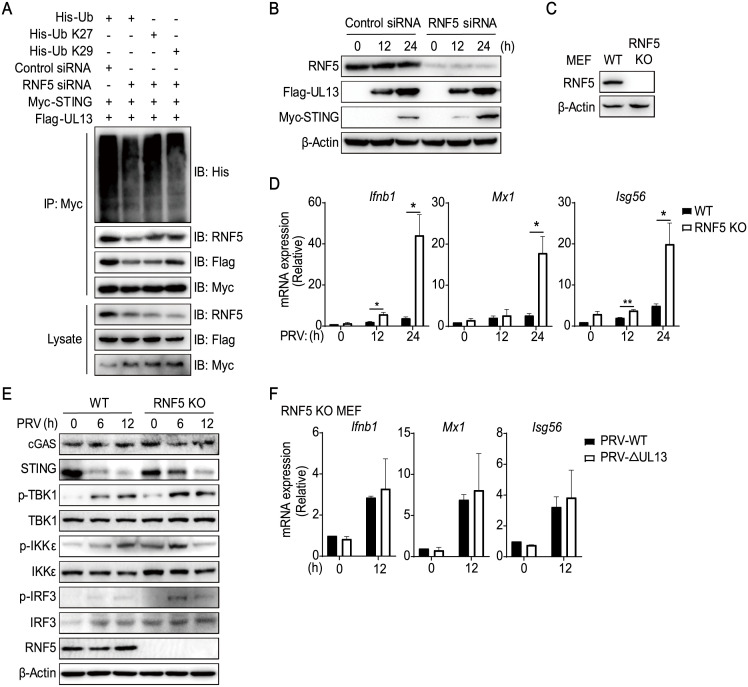
RNF5-deficiency potentiates PRV-induced antiviral immune responses. (A) Immunoblotting analysis of indicated proteins in immunoprecipitated samples and whole-cell lysates of HEK293T cells transfected with RNF5 siRNA or control siRNA (50 μM). Twenty-four hours after transfection, cells were further transfected with Flag-UL13, Myc-STING, and His-Ub for another 24 h before analysis. (B) Immunoblotting analysis of protein levels of RNF5, UL13, and STING in whole-cell lysates of HEK293T cells transfected with RNF5 siRNA or control siRNA for 24 h. Cells were then transfected with Flag-UL13 and Myc-STING for the indicated times (above lane). (C) Immunoblotting analysis of RNF5 protein expression in wild type (WT) and RNF5-deficient (RNF5 KO) MEFs. (D) qPCR analysis of transcription of *Ifnb1* and downstream genes (*Mx1*, *Isg56*) in WT and RNF5 KO MEFs infected with PRV (MOI = 1) for the indicated times. (E) Immunoblotting analysis of cGAS, STING, phosphorylated (Ser172) and total TBK1 and IKKε, phosphorylated (Ser396) and total IRF3, or RNF5 in whole-cell lysates of WT and RNF5 KO MEFs infected with PRV (MOI = 1) for the indicated times. (F) qPCR analysis of mRNA expression of *Ifnb1* and downstream genes (*Mx1*, *Isg56*) in RNF5 KO MEFs infected with PRV-WT or PRV-ΔUL13 (MOI = 1) for indicated times. Data are representative of two (A, C) or three (B, E) independent experiments, or are pooled from three independent experiments (D, F, mean ± SD). *p < 0.05, **p < 0.01 (Student’s *t* test).

Given that enforced expression of RNF5 catalyzed K27-/K29-linked ubiquitination of STING, we next addressed whether endogenous RNF5 promoted STING ubiquitination in the same way. To do this, we synthesized siRNA oligos that specifically target human RNF5 (RNF5 siRNA) [37] and then verified the knockdown efficiency of RNF5 siRNA in HEK293T cells (S5B Fig). Knockdown of RNF5 substantially attenuated K27-linked or K29-linked ubiquitination of STING related to UL13 as well as the interaction between UL13 and STING (Fig 6A), indicating that endogenous RNF5 is responsible for catalysis of STING ubiquitination. Similarly, knockdown of RNF5 also alleviated UL13-mediated STING degradation (Fig 6B and S5C Fig), confirming the bridge role of RNF5 in regulation of UL13-induced ubiquitination and degradation of STING. We next investigated whether endogenous RNF5 was involved in regulation of STING-mediated immune response induced by PRV in MEFs. Therefore, RNF5-deficient MEFs were generated by CRISPR/Cas9 technology. Gene mutation of *Rnf5* in RNF5-deficient MEFs was verified by PCR assay following DNA sequencing (S5D Fig) and lack of RNF5 protein in the cells was confirmed by immunoblotting (Fig 6C). Upon PRV infection, deficiency of RNF5 boosted induction of *Ifnb1* and ISGs (Fig 6D and S5E Fig), enhanced phosphorylation of TBK1, IKKε, and IRF3, and decelerated STING degradation in MEFs (Fig 6E and S5F Fig), which is specifically striking at 6 hours. These data show that RNF5 suppresses STING-mediated antiviral response during PRV infection. Moreover, induction of *Ifnb1* and downstream ISGs mediated by PRV-WT or PRV-ΔUL13 were similar in RNF5-deficient MEFs, further supporting that UL13-mediated suppression of antiviral responses is dependent on RNF5 (Fig 6F). Altogether, these results suggest that RNF5 is critical for UL13-mediated STING ubiquitination and degradation and activation of STING-mediated downstream signaling process.

### UL13 facilitates immune evasion of PRV *in vivo*

**Fig 7:**
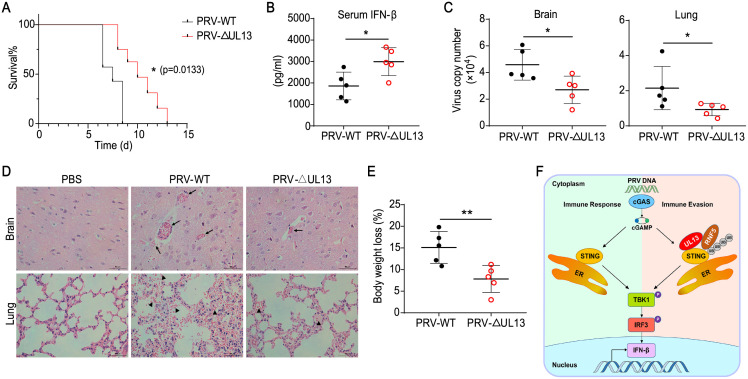
UL13 deficiency enhances host defense against PRV infection *in vivo*. (A) Survival rate of C57BL/6 mice (n = 7 in each group) infected with a lethal dose (0.5 ×10^6^ PFU, the same below) of PRV-WT or PRV-ΔUL13. (B) ELISA of IFN-β in serum from mice infected with a lethal dose of PRV-WT or PRV-ΔUL13 (n = 5 in each group). Serum was collected 24 h post-infection. (C) qPCR analysis of viral propagation in tissue of mouse brain and lungs at day 6 after infection with a lethal dose of PRV-WT or PRV-ΔUL13 (n = 5 in each group). (D) Hematoxylin and eosin (H&E) staining of sections of brain and lung of mice as in (C); arrows indicate cerebral vascular congestion and hemorrhage in brain; triangles indicate diffuse alveolar hemorrhage or alveolar exudates. Scale bars, 50 μm. Original magnification, ×40. (E) Body weight loss of mice infected with PRV-WT or PRV-ΔUL13 as in A at day 6. (F) A working model of how UL13 regulates the STING-mediated immune response. Data are representative of two independent experiments (A, D) or one independent experiment (B, C, E; mean ± SD). *p < 0.05, **p < 0.01 (Student’s unpaired *t* test or log-rank test in A).

Next, we investigated the functions of UL13 in PRV immune evasion *in vivo*. To know the role of UL13 in PRV pathogenicity, we intraperitoneally infected mice with a lethal dose (0.5×10^6^ PFU) of wild-type PRV and PRV-ΔUL13 respectively. We found that mice infected with PRV-ΔUL13 had enhanced survival relative to mice infected with wild-type PRV (Fig 7A), which indicated that deficiency of UL13 strengthened host defense against PRV. Indeed, levels of IFN-β in serum from mice infected with PRV-ΔUL13 were significantly higher than those of mice infected with wild-type PRV (Fig 7B). Meanwhile, the virus copies in the brain and lung from mice infected with PRV-ΔUL13 were lower compared to those in PRV-infected mice (Fig 7C). Consequently, mice infected with wild-type PRV exhibited histopathological changes including acute viral encephalitis characterized by cerebral vascular congestion and hemorrhage in brain and interstitial pneumonia or hemorrhagic pneumonia in lung while mice infected with PRV-ΔUL13 showed mild pathological changes (Fig 7D), suggesting that UL13 contributes to PRV pathogenicity *in vivo*. In addition, the body weight of mice infected with PRV-ΔUL13 had less loss than that of mice infected with wild-type PRV (Fig 7E). In addition, the basal replication of PRV-WT and PRV-ΔUL13 were similar in both IFNAR-deficient BMDMs and STING-deficient PK15-cells (S5G–S5I Fig), demonstrating that IFN or STING-mediated immune response is responsible for attenuation of mutant PRV. Collectively, these data suggested that UL13 suppressed host antiviral responses in response to PRV infection and exacerbated the PRV-mediated pathogenicity *in vivo* (Fig 7F).

## Discussion

The innate immune system is the first line of host defense against pathogenic invasion, and type I IFN is a pivotal cytokine for early against of viral infection. To successfully establish and maintain infection, herpesviruses, such as HSV-1 and HCMV, have evolved various strategies to evade host antiviral innate immunity and facilitate viral infection and replication. In contrast to other herpesviruses, studies about PRV proteins involved in regulation of the cGAS-STING signaling pathway are limited [20]. Here we identified PRV UL13 as an inhibitor for STING-mediated innate antiviral response via targeting STING. Expression of UL13 decreases STING protein expression and thus suppresses DNA virus- or cytosolic DNA-induced antiviral immune response of host. Conversely, deficiency of UL13 enhances STING-mediated signaling and increases transcription of downstream antiviral factors. Thus PRV UL13 functionally acts as an antagonist of DNA-virus-triggered innate immune response. In consistent with our observation, recent studies also reported UL13 acted as a suppressor of innate immunity via promoting degradation of IRF3 or quickening degradation of a TBK1/IKKε binding protein peroxiredoxin 1 (PRDX1) [38–40]. However, whether UL13 directly or indirectly ubiquitinates IRF3 or PRDX1 remains unclear. In addition, a previous study also indicated that UL13 did not alter IRF3 protein levels but suppressed IRF3 binding activity [38,40]. Thus, the functional roles of UL13 on IRF3 are still obscure. In contrast, we found that UL13 promotes ubiquitination and degradation of STING, the key upstream adaptor protein of TBK1 and IRF3, thereby impairing the phosphorylation of IRF3 and TBK1. Additionally, we also identified that UL13 partnered with the E3 ligase RNF5 to accelerate ubiquitination of STING, supporting that UL13 does not catalyze ubiquitination of STING directly. Hence, our finding demonstrates a novel distinct mechanism of UL13 acting as a suppressor of innate antiviral responses via targeting protein stability of STING, highlighting the importance of UL13 in regulation of cGAS-STING-mediated signaling pathway.

STING biological function is regulated by a variety of post-translation modifications including phosphorylation and ubiquitination of the protein that affect its signaling functions. For example, it has been reported that E3 ubiquitin ligases TRIM56, TRIM32, and MUL1 promote the K63-linked ubiquitination of STING [41–43]. RNF26 promotes K11-linked polyubiquitination of STING and AMFR facilitates the K27-linked polyubiquitination of STING [44,45]. On the other hand, E3 ubiquitin ligases RNF5, TRIM29, and TRIM30α, are accountable for regulation of protein stability of STING, which mainly promotes K48-linked polyubiquitination of STING [33–35,46]. These studies suggest that STING can undergo multiple ubiquitination modifications. Here we show that PRV UL13 interacts with STING and promotes degradation of STING to impair its protein stability. Interestingly, UL13-induced STING degradation is not dependent on increasing the canonical K48-linked polyubiquitination of STING. Instead, UL13 promotes the K27-/K29-linked ubiquitination of STING to facilitate its degradation. Our results thus identified a previously unappreciated mechanism for regulation of STING stability. In fact, we identified 6 critical residue sites of STING that were responsible for K27-/K29-linked form by UL13. At least the lysine residue 150 of STING could undergo four different types of ubiquitination including K11, K27, K48, K63-linked ubiquitination [33,41,45,47]. Our results further indicate that multiple lysine residues of STING can undergo ubiquitination to modulate the function of STING.

The E3 ubiquitin ligase RNF5 has been shown to promote K48-linked ubiquitination and degradation of STING and MAVS, thereby suppressing the host antiviral immune responses [33,37]. Notably, the RNF5-mediated K48-linked ubiquitination of STING has happened during RNA virus infection [33]. So far, whether RNF5 induces ubiquitination of STING during DNA virus infection remains unclear. Here, we showed that PRV UL13 recruits RNF5 to mediate STING degradation through induction of K27-/K29-linked ubiquitination of STING, but not via canonical K48-linked ubiquitination of STING. These results demonstrate that RNF5 can modulate different types of ubiquitination of STING in response to infection of distinct viruses. Notably, previous studies have shown that RNF5 can mediate K27-linked, K29-linked, or K48/K33-linked ubiquitination [48–50]. Hence, our results and those of others suggest that RNF5 can mediate multiple types of ubiquitination to regulate antiviral immunity or other biological processes and expand the role of RNF5 in regulation of DNA virus-triggered antiviral immune responses.

In herpesviruses, the UL13 and US3 are two protein kinases that mediate viral gene expression and replication. Numerous studies have shown that herpesvirus US3 negatively regulates antiviral immune responses through multiple mechanisms [23,51]. Herpesvirus UL13 is packaged in the tegument and is proposed to phosphorylate itself and some viral proteins [52]. Interestingly, recent studies also suggested that PRV UL13 was a novel viral immune escape protein [38,39], however, the mechanisms of UL13 in regulation of antiviral immune response remain unclear. Our results showed that UL13 targets STING to inhibit type I interferon signaling pathway, suggesting that UL13 not only processes viral proteins but also regulates the host antiviral response. Accordingly, UL13 could be a potential novel target for generation of effective attenuated live vaccines for Pseudorabies. In fact, there have been frequent outbreaks of pseudorabies caused by variant PRV in China since 2011 and the classical attenuated vaccine (strain Bartha-K61) might not provide sufficient protection against variant PRV infection [53]. Moreover, UL13 is conserved in herpesviruses and has homologs among the alpha-, beta-, and gamma-herpesviruses [54]. Further studies need to be performed to determine whether other herpesvirus UL13 homologs also antagonize antiviral responses. In summary, we have identified that PRV UL13 as a suppressor of host innate immune response via targeting STING-mediated signaling through recruitment of E3 ligase RNF5 to impair the stability of STING protein. Our results thus demonstrate a novel function of PRV tegument protein UL13 in regulation of host antiviral response and provide a potential mechanism for PRV immune escape.

## Materials and methods

### Ethics statement

All animal protocols were reviewed and approved by Shandong Agricultural University Animal Care and Use Committee (Approval Number: # SDAUA-2018-057) and were performed in strict accordance with the Animal Ethics Procedures and Guidelines of the People’s Republic of China.

### Cells and viruses

HEK293T cells, PK-15 cells, MEFs, Hela cells, and BHK-21 cells were purchased from the American Type Culture Collection (ATCC, VA, USA). Cells were maintained in Dulbecco’s minimal essential medium (DMEM) (Gibco, USA) containing 10% fetal bovine serum (FBS) (Biological Industries, Israel) and 1% penicillin-streptomycin (Gibco) at 37°C and 5% CO_2_. IFNAR-deficient BMDMs were provided by Dr. Feng Ma (SuZhou Institute of Systems Medicine, SuZhou, China) and were cultured as previously described [55]. All cells were tested negative for mycoplasma. PRV (Bartha-K61 strain) was purchased from China Veterinary Culture Collection Center (Cat#CVCC AV249, Beijing, China) and was purified in BHK-21 cells.

### Generation of UL13-deficient PRV

The PRV-ΔUL13 recombinant strain was generated according to methods described previously [56]. Two gRNAs targeting the PRV UL13 gene were designed using the gRNA website (https://zlab.bio/guide-design-resources) and were synthesized (Sango Biotech, Shanghai, China). One gRNA sequence was first ligated to pX459M plasmid and another one was ligated into EZ-GuideXH plasmid for gRNA transferring. The plasmids were then digested by Xho I and Hind III enzymes. Finally, the two gRNAs were ligated into pX459M plasmids. UL13 gene was amplified by PrimeSTAR HS DNA Polymerase with PRV (strain Bartha-K61) genomic DNA as a template, followed by BamH I and Hind III digestion and were ligated with pEGFP-C1 vector to construct an EGFP-UL13 donor plasmid. HEK293T cells were transfected with EGFP-UL13 donor and pX459M-EZ-UL13-gRNA1/2 plasmids for 24 h. Cells were infected with PRV for 24 h (MOI = 1). The virus mixture was collected and subjected to the plaque assay. Monoclonal viruses expressing green fluorescence were singled out by fluorescence microscopy (Nikon, Japan) and were identified by PCR following DNA sequencing. The UL13 gRNA target sequences are: #1 5’-GAGCACGCTGCCGTACGATC-3’; #2 5’-GGCGATCGACCTGTGCGCGC-3’. All viruses were amplified and titrated in PK-15 cells using standard protocols.

### Plasmids

PRV tegument genes were amplified from PRV (strain Bartha-K61) genomic DNA by PrimeSTAR HS DNA Polymerase and cloned into pCMV6-XL4 (OriGene, USA) or pCAGGS (Biovector, China) expression vectors. The UL13 with N-terminal Flag tag was obtained from pCMV6-XL4-UL13 vector as a template, followed by EcoR I and Not I digestion and were cloned into a lentivirus-based vector pCDH-CMV-MCS-EF1-Puro (Addgene, USA). shRNA oligos targeting the PRV UL13 gene were designed using the siRNA website (http://sirna.wi.mit.edu/) and were synthesized by Sango Biotech, China. The shRNA oligos were then ligated to pLKO.1-puro plasmid (Addgene) followed by EcoR I and Age I digestion sites. Human STING and its truncation mutants (amino acids 1–139, amino acids 140–380, amino acids 140–340, amino acids 180–380, amino acids 180–340, and amino acids Δ180–340) were amplified from cDNA of Hela cells, followed by EcoR I and Not I digestion and were ligated with pCMV-Myc vector (Addgene). Ubiquitin mutants (K6, K11, K27, K29, K33, K48, K63, and AKR) or STING ubiquitin mutants (K20, K137, K150, K224, K236, K289, K338, K347, K370, and STING-AKR) were generated by using a Site-Directed Mutagenesis Kit according to the manufacturer’s protocol (New England Biolabs, USA). TBK1 and IRF3-5D plasmids were described previously [57]. All constructs followed standard molecular cloning protocols and then were sequenced. Protein expression of the constructs was further confirmed by immunoblotting.

### Antibodies and reagents

The antibodies used and the sources are as follows: Antibodies against TBK1 (1:1,000, 3013), p-TBK1 (1:1,000, 5483), IKKε (1:1,000, 3416), p-IKKε (1:1,000, 8766), p-IRF3 (1:500, 4947) were purchased from Cell Signaling Technology. Antibodies against cGAS (1:2,000, 26416-1-AP), STING (1:2,000, 19851-1-AP), HA tag (1:2,000, 51064-1-AP), His tag (1:2,000, 66005-1-lg), and β-actin (1:20,000, 66009-1-lg) were purchased from Proteintech Group Inc. IRF3 antibody (1:1,000, sc-33641), RNF5 antibody (1:1,000, sc-81716), and c-Myc antibody (1,1,000, sc-40) were obtained from Santa Cruz Biotechnology. Monoclonal anti-Flag (1,1,000, F1804) antibody was purchased from Sigma-Aldrich, Inc. Anti-Calreticulin antibody (1,100, ab2907) was purchased from Abcam. Poly(dA:dT) (tlrl-patn) and cGAMP (tlrl-nacga 23–02) were purchased from Invivogen. Lipofectamine 2000 transfection reagent and Opti-MEM were from Thermos Fisher Scientific. Protease inhibitors were purchased from Roche. PMSF was purchased from Solarbio Life Sciences, Beijing, China.

### Daul-luciferase reporter assays

Human IFN-β and ISRE reporter plasmids were described previously [58]. PK-15 cells, UL13-PK-15 cells were cotransfected with the IFN-β or ISRE luciferase reporter plasmids and expression plasmids encoding key PRV tegument proteins or expression plasmids encoding STING, TBK1, IRF3-5D, or empty control vectors by using Lipofectamine 2000 (Invitrogen, USA). Twenty-four hours after transfection, cells were stimulated with poly(dA:dT) (2 μg/ml) for 12 h or directly harvested for luciferase assay. Cell lysates were prepared and analyzed using the Dual-Luciferase Report Assay System (Promega, USA) according to the manufacturer’s instructions. The renilla luciferase reporter gene (pRL-TK, Promega) was used as an internal control.

### Lentivirus infection and stable cell line generation

For generation UL13 expression stable cell line, lentiviral particles were produced in HEK293T cells transfected with two packaging plasmids (psPAX2 and pVSVG) and empty vector or pCDH-Flag-UL13 plasmid using Lipofectamine 2000 (Invitrogen). After 24 h, the recombinant viruses were filtered, then infected PK-15 cells and supplemented with polybrene (6 μg/ml, Cat#H8641, Solarbio, China). Cells were selected with puromycin (Cat#IP1160, Solarbio) at a final concentration of 14 μg/ml for 5 days. Monoclonal cells were obtained by the limited dilution method in 96-well plates and determined by immunoblotting with Flag antibody (Sigma). For generation of UL13 RNAi stable cells, lentiviral particles were produced in HEK293T cells transfected with two packaging plasmids (psPAX2 and pMD2.G) and control or pLKO.1-UL13-RNAi plasmids using Lipofectamine 2000 (Invitrogen). After 24 h, the recombinant viruses were filtered, then infected MEFs and supplemented with polybrene (6 μg/ml). Cells were selected with puromycin at a final concentration of 3 μg/ml for 3 days. Monoclonal cells were obtained by the limited dilution method in 96-well format. UL13-shRNA MEFs were verified by qPCR. The following sequences were targeted for UL13 mRNA: #1 5’-TTAGCCTCATGGCCCTCAACT-3’; #2 5’-GCAACATCTTTGTGCGCACGT-3’. The control sequence (5’-GGAAGATGTATGGAGACATGG-3’) was followed previously described [13,25,28].

### Coimmunoprecipitation and immunoblotting

Immunoprecipitation and immunoblotting were performed as previously described [12,58]. Briefly, indicated cells were transfected with pCMV6-Flag-UL13, pCAGGS-HA-RNF5, pCAGGS-HA-TRIM21, pCMV-Myc-STING mutants or His-Ubiquitin mutants using Lipofectamine 2000 (Invitrogen). Twenty-four hours after transfection, cells were lysed on ice with a lysis buffer (50 mM Tris-Cl at pH 7.4, 150 mM NaCl, 1% Triton X-100, 1% sodium deoxycholate, 1 mM Na_3_VO_4_, 1 mM EDTA, and 1 mM PMSF) for 60 min. Extracts were immunoprecipitated with 1 μg of indicated antibodies and protein A/G PLUS-Agarose beads (sc-2003; Santa Cruz Biotechnology). Whole-cell lysates or immunoprecipitated extracts were then separated by sodium dodecyl sulfate polyacrylamide gel electrophoresis (SDS-PAGE) and transferred to a polyvinylidene fluoride membrane (Millipore) for immunoblotting with specific antibodies.

### DNA quantification and polymerase chain reaction (PCR)

MEFs, IFNAR-deficient BMDMs, or STING-deficient PK-15 cells were infected with PRV-WT or PRV-ΔUL13 (MOI = 1) for indicated periods. Total DNA was extracted using a DNA isolation kit (Cat#DP304, TIANGEN, China) following the manufacturer’s protocol. PRV copy number was quantified by qPCR using gD gene primers [59]. Regular PCR assay was performed by using PrimeSTAR HS DNA Polymerase (Takara Bio, Beijing, China), and the PCR products were analyzed by gel electrophoresis with 1% agarose gel and observed under UV light (Alpha RED, ProteinSimple).

### Reverse transcription and quantitative real-time PCR (qPCR)

Total RNA was extracted by SV Total RNA Isolation System (Promega) and was reversely transcribed to cDNA using M-MLV reverse transcriptase with RNase inhibitor (Takara Bio, Beijing, China). qPCR was performed in triplicated determinants with RealStar Green Fast Mixture (A303, GenStar) on a StepOne plus thermal cycler (ABI, Thermo Fisher Scientific). Threshold cycle numbers were normalized to triplicated samples amplified with primers specific for glyceraldehyde-3-phosphate dehydrogenase (*GAPDH*). qPCR primer sequences are listed in S1 Table.

### Immunofluorescence

Hela cells were transfected with the indicated plasmids and then fixed for 20 min in 4% paraformaldehyde. Cells were then permeabilized for 10 min with 0.1% Triton X-100 and then blocked with 5% bovine serum albumin (BSA, Sigma) for 30 min. Cells were incubated with the appropriate primary antibodies for 120 min and then stained with Alexa Fluor 488- or 594- conjugated secondary antibodies (Proteintech Group Inc, China) for 60 min. Images were acquired using a laser scanning confocal microscope with LAS X software (Leica, Germany).

### Nano LC-MS/MS

HA-STING and Flag-UL13 expression plasmids were cotransfected into HEK293T cells with Lipofectamine 2000 (Invitrogen). Cells were harvested 24 h after transfection, and the lysates were immunoprecipitated with anti-HA antibody. After washing, the eluted samples were resolved with SDS-PAGE, followed by Coomassie brilliant blue staining. Nano LC-MS/MS analysis were performed at the Technological Platform of Mass Spectrum Center of Institute of Microbiology, Chinese Academy of Sciences (LCQ Deca XP Plus, Thermo Fisher Scientific). Specifically, the protein bands were cut from the gel and digested by trypsin to generate peptide mixture and dried by Speed-vac. The dried peptides samples were resuspended in a solvent of 0.1% formic acid (v/v) and subjected to an EASY-nLC 1000 interfaced via a Nanospray Flex ion source to an Orbitrap Fusion Tribrid mass spectrometer (Thermo Fisher Scientific). The peptides were loaded onto a trap column (C18, 3 μm particles, 100μm ID, 3 cm length, Dr. Maisch GmbH) and separated using an analytical column (C18, 1.9 μm particles, 150 μm ID, 15 cm length, Dr. Maisch GmbH) at a flow rate of 400 nL/min with a 60 min LC gradient composed of Solvent A (0.1% formic acid (v/v)) and Solvent B (acetonitrile, 0.1% formic acid (v/v)). The gradient was 3–8% B for 5 min, 8–20% B for 40min, 20–35% B for 10 min, 35–80% B for 3 min, and finally 80% B for 2 min. The mass spectrometer was operated in a data-dependent acquisition mode, in which the precursor MS1 scan (m/z 350–1550) was acquired in the Orbitrap at a resolution setting of 120,000, followed by Orbitrap HCD-MS/MS and ITHCD-MS/MS of the 20 most abundant multiply charged precursors in the MS1 spectrum.MS2 spectra were acquired at a resolution of 30,000.

MS/MS data was processed using Mascot search engine (v.2.5.1, 2014, http://www.matrixscience.com; Matrix Science Ltd., London, UK). Tandem mass spectra were searched against SWISS-Prot/TrEMBL (http://www.expasy.org/), and Trypsin/P was specified as cleavage enzyme allowing up to 2 missing cleavages. For precursor ions, the mass error was set to 10 ppm, and for fragment ions, the mass error is set to 0.02 Da. Carbamidomethylation on Cys was specified as fixed modification. Oxidation on Met was specified as variable modification. False discovery rate (FDR) thresholds for protein, peptide, and modification site were adjusted to < 1%. P value < 0.05 was considered statistically significant. P-values were corrected for FDR in each dataset. All the other parameters in Mascot were set to default values.

### RNA-mediated interference (RNAi)

siRNA specifically targeting human RNF5 and non-targeting control siRNA were reported previously [37]. siRNA oligos were synthesized by Shanghai GenePharma Co., Ltd, China, and were transfected into HEK293T cells using Lipofectamine RNAiMAX (Invitrogen) reagent according to the manufacturer’s instructions at a final concentration of 50 nM.

### Generation of RNF5 and STING knockout cells

For generation of RNF5 knockout cells, custom gRNAs targeting mouse Rnf5 exon 3 and exon 4 were designed using the gRNA website (https://zlab.bio/guide-design-resources) and were synthesized (Sango Biotech, Shanghai, China). The gRNA sequences were ligated to pX459M plasmid or EZ-GuideXH plasmid, respectively, and were then digested by Xho I and Hind III enzymes. Finally, the two gRNAs were ligated into pX459M plasmids to generate pX459M-EZ-Rnf5-gRNA1/2 plasmid containing two different gRNAs. MEFs were transfected with pX459M-EZ-Rnf5-gRNA1/2 plasmid or control vector by using Lipofectamine 2000. At 24 h after transfection, cells were screened with puromycin (3 μg/ml) for 3 days. Monoclonal cells were obtained by the limited dilution method in 96-well format and deficiency of RNF5 was verified by regular PCR following DNA sequencing and immunoblotting. The Rnf5 gRNA target sequences are: gRNA-1: 5’-TCGTCCCTCTTTATGGGCGA-3’; gRNA-2: 5’-AGTAGTAGCCCTGCTCGTCG-3’. For generation of STING-deficient cells, custom gRNAs targeting pig STING exon 4 and exon 7 were designed, synthesized and constructed as above mentioned. PK-15 cells were transfected with pX459M-EZ-STING-gRNA1/2 plasmid or control vector by using Lipofectamine 2000. At 24 h after transfection, cells were screened with puromycin (14 μg/ml) for 3 days. Monoclonal cells were obtained by the limited dilution method in 96-well format and deficiency of STING protein was verified by immunoblotting. The STING gRNA target sequences are: gRNA-1: 5’-AGCCACCGGAGCGTGTATTC-3’; gRNA-2: 5’- GCGCCACAAGAACGTACTCG-3’.

### Animal experiments

C57BL/6 mice were purchased from Beijing Vital River Laboratory Animal Technology Co., Ltd, and mice with 6 weeks of age were used for all experiments. All mice were maintained in pathogen-free barrier facilities. For survival assay, mice were intraperitoneally inoculated with 0.2 ml of 0.5×10^6^ PFU of PRV-WT or PRV-ΔUL13 (n = 7 in each group) as previously described [60]. The survival time of mice in each group was recorded. For *in vivo* infection, mice were intraperitoneally inoculated with 0.2 ml of 0.5×10^6^ PFU of PRV-WT, PRV-ΔUL13, or 0.2 ml of PBS as uninfected controls (n = 5 in each group). The mouse serum was collected at 1 dpi. The lungs and brains of infected-mice were collected at 6 dpi for detection of viral load or stained with hematoxylin and eosin (H&E).

### Enzyme-linked immunosorbent assay (ELISA)

IFN-β secretion in serum was quantified by using paired anti-mouse IFN-β antibodies (Capture antibody, 22400–1; Detection antibody, 32401–1; PBL) according to the manufacturer’s instructions. Mouse IFN-β (12405–1, PBL) was used as protein standard.

### Histopathology

Histopathology was performed as previously described [59]. In brief, tissues from mouse brain or lung were fixed overnight in 10% neutral-buffered formalin, trimmed, and embedded in paraffin. Sections were cut into 5 μm and stained with hematoxylin-eosin (H&E). Images were acquired on an Olympus microscope (CX41RF) using imaging software (MiE V3.1).

### Statistical analysis

P values were calculated with a two-tailed paired or unpaired Student’s *t* test by Prism GraphPad software (v8.0). P values of ≤0.05 were considered significant. For mice survival study, statistical significance was analyzed with the log-rank test.

## Supporting information

S1 Fig PRV UL13 inhibits cGAS-STING-induced expression of ISGs in PK-15 cells. (A) Luciferase activities in PK-15 cells cotransfected with IFN-β-luc reporter or ISRE-luc reporter and key PRV tegument protein expression plasmids or control empty vector as indicated. Twenty-four hours after transfection, cells were stimulated with B-DNA (2 μg/ml) for 12 h, and cell lysates were analyzed for luciferase activity. (B and C) qPCR analysis of mRNA expression of ISGs (*MX2*, *ISG15*, *ISG54*, *OAS1b*) in UL13-PK-15 cells or control cells transfected with B-DNA (1 μg/ml) (B) or cGAMP (1 μg/ml) (C) for the indicated times. (D, E) Cumulative results for densitometric quantitation of indicated proteins (above) in Fig 1D and 1E as normalized to the levels of β-actin loading control. Data are pooled from three independent experiments (A-E, mean ± SD). *p < 0.05, **p < 0.01 (Student’s *t* test).
(EPS)

S2 Fig UL13 deficiency increases PRV-induced expression of ISGs in MEFs. (A) Immunoblotting analysis of UL13 protein expression in HEK293T cells cotransfected with Flag-UL13 and UL13-RNAi or control RNAi plasmids for 24 h. (B) qPCR analysis of gene transcription of UL13 in HEK293T cells transfected with UL13-RNAi plasmids and control RNAi plasmid. 24 h after transfection, cells were infected with PRV (MOI = 1) for 24 h. (C) qPCR analysis of PRV UL13 gene expression in UL13-RNAi stable mouse embryonic fibroblasts (MEFs) and control cells infected with PRV (MOI = 1) infection for 24 h. (D) Cumulative results for densitometric quantitation of indicated proteins (above) in Fig 2B as normalized to the levels of β-actin loading control. (E) PCR analysis of UL13 gene in PRV-WT and PRV-ΔUL13. (F) qPCR analysis of PRV-WT (MOI = 1) or PRV-ΔUL13 (MOI = 1) replication for the indicated times. (G) qPCR analysis of ISGs (*Mx2*, *Isg15*, *Oas1b*) mRNA expression in MEFs infected with PRV (MOI = 1) or PRV-ΔUL13 (MOI = 1) for the indicated times. (H) Cumulative results for densitometric quantitation of indicated proteins (above) in Fig 2D as normalized to the levels of β-actin loading control. Data are representative of two independent experiments (A-C, E) or pooled from three independent experiments (D, F-H, mean ± SD). *p < 0.05, **p < 0.01 and ***p < 0.001 (Student’s *t* test).
(EPS)

S3 Fig UL13 does not regulate gene expression of STING. (A) qPCR analysis of *STING* mRNA expression in PK-15 cells stably expressing UL13 and control cells. (B) qPCR analysis of *Sting* mRNA expression in MEFs infected with PRV-WT (MOI = 1) or PRV-ΔUL13 (MOI = 1) for the indicated times. Data are pooled from three independent experiments (A, B, mean ± SD). ns, not significant (Student’s *t* test).
(EPS)

S4 Fig Association of UL13 with STING. (A) Immunoblotting (IB) analysis of indicated proteins in immunoprecipitated (IP) samples and whole-cell lysates of HEK293T cells transfected with Flag-UL13 and HA-STING. Anti-Flag immunoprecipitates were analyzed by immunoblotting with an anti-HA antibody. Levels of the transfected proteins were analyzed by immunoblotting with anti-HA or anti-Flag antibodies. (B) Colocalization of UL13 and STING in Hela cells. Hela cells were transfected with Flag-UL13 and HA-STING as indicated for 24 h before confocal microscopy. Scale bars, 10 μm. Data are representative of two independent experiments (A-B).
(EPS)

S5 Fig Lack of RNF5 promotes PRV-induced expression of ISGs in MEFs. (A) LC-MS analysis of E3 ligase in immunoprecipitated samples of HEK293T cells transfected with Flag-UL13 and HA-STING. Cells were co-transfected with HA-STING and Flag-UL13 expression plasmids for 24 h and anti-HA immunoprecipitates were subjected to LC-MS analysis. (B) qPCR analysis of *RNF5* mRNA expression in HEK293T cells transfected with RNF5 siRNA or control siRNA at different doses for 24 h. (C) Cumulative results for densitometric quantitation of indicated proteins (above) in Fig 6B as normalized to the levels of β-actin loading control. (D) PCR analysis of Rnf5 gene in WT and RNF5-deficient MEFs. (E) qPCR analysis of ISGs (*Mx2*, *Isg15*, *Oas1b*) mRNA expression in WT and RNF5-deficient MEFs infected with PRV-WT (MOI = 1) for the indicated times. (F) Cumulative results for densitometric quantitation of indicated proteins (above) in Fig 6E as normalized to the levels of β-actin loading control. (G) qPCR analysis of *Ifnar* mRNA in wild type (WT) and IFNAR-deficient (IFNAR KO) BMDMs. (H, I) qPCR analysis of gD gene expression in IFNAR-deficient BMDMs (H) or STING-deficient PK-15 cells (I) infected with PRV-WT or PRV-ΔUL13 (MOI = 1) for indicated times. Data are representative of one experiment (A, B, D, G) or pooled from three independent experiments (C, E, F, H, I, mean ± SD). *p < 0.05, **p < 0.01 and ***p < 0.001, ns, not significant (Student’s *t* test).
(EPS)

S1 Table Primers used in this study. (DOCX)
