# Supplementary material for: Pseudorabies virus tegument protein UL13 recruits RNF5 to inhibit STING-mediated antiviral immunity
Source: PLoS Pathog. 2022 May 18;18(5):e1010544. doi: 10.1371/journal.ppat.1010544 (PMC9154183; doi:10.1371/journal.ppat.1010544)
Supplement: S1 Table — (DOCX) [file ppat.1010544.s006.docx]

**S1 Table: Primers used in this study.**

| **Gene** | **Forward sequence (5’–3’)** | **Reverse sequence (5’–3’)** |
| --- | --- | --- |
|  | **Primer sequences for regular qPCR** | |
| m*GAPDH* | ATCAAGAAGGTGGTGAAGCA | AGACAACCTGGTCCTCAGTGT |
| m*Ifnb1* | CAGATCACCCAGAAGATCG | CCCTTGTTATTCCTCACCAG |
| m*Mx1* | GGCAGACACCACATACAACC | CCTCAGGCTAGATGGCAAG |
| m*Mx2* | ACCAGAGTGCAAGTGAGGAGCT | GTACTAGGGCAGTGATGTCCTG |
| m*Isg15* | GGGGGAGTATGGCCTAAAGC | CCAACACTGGCTCTGGATGG |
| m*Isg56* | TACAGGCTGGAGTGTGCTGAGA | CTCCACTTTCAGAGCCTTCGCA |
| m*Oas1b* | TTTCTCACAGCCTGCTTTATC | TCCTAACAGTGCCATTCCCT |
| m*Sting* | TAGCCTCCTGACTGAGCCTT | CAGGTCAACAGAGAGCAGGG |
| m*Ifnar*  p*GAPDH* | AAAGACGAGGCGAAGTGGTT  TACACTGAGGACCAGGTTGTG | TTGTCCGGGAGGAGAGATGT  TTGACGAAGTGGTCGTTGAG |
| p*IFNB1* | TGCATCCTCCAAATCGCTCT | ATTGAGGAGTCCCAGGCAAC |
| p*MX1* | GCTTTCAGATGCTTCGCAGG | TGTCGTATGGCTGATTGCCT |
| p*MX2* | ATCAGCTACCGCAAAGTGGAG | TACTTCCTGATGAGCGCCTTG |
| p*ISG15* | GCCTTCCAGCAGCGTCT | GCGTTGCTGCGACCCT |
| p*ISG54* | GCACAGCAATCATGAGTGAGAC | CTGGCCCCTGCAGTCTTTTA |
| p*ISG56* | TCCGACACGCAGTCAAGTTT | TGTAGCAAAGCCCTGTCTGG |
| p*OAS1b* | CAGGCCAACAGGTTCAGACAG | CAGGAAACCGCAGACGATGT |
| p*STING* | CTGCTGCTGTCCTGCTACTT | TTGCAGAGACTTCAGCTGGG |
| h*RNF5* | AACGGCAAGAGTGTCCAGTA | TTAATCTGGGATCCTGGGGC |
| PRV *UL13* | CGGGGACTTTAGCCTCATGG | TAAAGTCCATCAGCACCCGC |
| PRV *gD* | CACGGAGGACGAGCTGGGGCT | GTCCACGCCCCGCCTGAAGCT |
|  | **Primer sequences for regular PCR** | |
| PRV *UL13* | ATGGCTGCTGGAGGA | TCAGGCAGCGAGTTC |
| m*Rnf5* | AGTCGTCCAACCGAGAGAGT | CCCACTCAGACATGAACCCC |
